# *In Vitro* and *In Vivo* Antioxidant and Anticancer Potentials of Royal Jelly for Dimethylhydrazine-Induced Colorectal Cancer in Wistar Rats

**DOI:** 10.1155/2022/9506026

**Published:** 2022-07-21

**Authors:** Maede Shakib Khoob, Seyed Mohammad Hosseini, Sohrab Kazemi

**Affiliations:** ^1^Department of Pathology, Babol Branch, Islamic Azad University, Babol, Iran; ^2^Cellular and Molecular Biology Research Center, Health Research Institute, Babol University of Medical Sciences, Babol, Iran

## Abstract

**Methods:**

This study was conducted among 60 rats, and groups consist of control, three separate groups for RJ, dimethylhydrazine (DMH), and vitamin E, and two separate treated groups with DMH + RJ and DMH + vitamin E. Additionally, the cytotoxicity of royal jelly was examined on HT-29 cell line. *Findings*. Based on the *in vitro* assessment using MTT assay, the LC50 of royal jelly was 1.781 mg/ml, and the highest cytotoxicity was observed at 25 mg/ml concentration after 48 hours. Meanwhile, in the *in vivo* study, after the 13th week, compared to the DMH group, the rats exposed to DMH + royal jelly experienced a significant less oxidative stress (*P* < 0.05) and a significantly greater total antioxidant capacity (TAC) level (*P* < 0.05). The expression of proliferating cell nuclear antigen (PCNA), platelet-derived growth factor (PDGF), and carcinoembryonic antigen (CEA) proteins significantly decreased among the animals receiving DMH + royal jelly compared to the DMH group. The pathological examinations revealed less congestion, necrosis, inflammation, and cell proliferation in the colon tissue of the RJ-treated group than that of the DMH group. Overall, the biochemical indices were better in the treatment groups in comparison with the DMH group.

**Conclusion:**

The results represented the clinical usability of royal jelly, as a substance with anticancer properties, to prevent and treat colorectal cancer. This issue is related to its effective antioxidant potential, which even exhibits more effectiveness than the vitamin E, which is known as a strong antioxidant.

## 1. Introduction

Cancer is among the conventional causes of the disease-caused death in the world [1]. Regarding colorectal cancer (CRC), a rise in reactive oxygen species (ROS) value plays a critical role in the development of the disease. According to Guéraud [2], a high ROS level influences the different signaling pathways related to proliferation, tumor survival, invasion, and metastasis. The body uses various mechanisms for adjusting the ROS concentration like the antioxidant-based enzymatic system. In addition, peptic ulcers, necrosis, and inflammations are closely associated with the cancers [3]. Lipid peroxidation products have been introduced as the oxidative stress biomarkers to determine the cell injury extent. Several researchers have utilized dimethylhydrazine (DMH) and its metabolites as toxic compounds for inducing the inflammation, precancer phases, and cancer lesions, especially in colon tissue [4]. The lesions include the different levels of necrosis and inflammation up to the cancer [5–7]. The DMH mostly affects by methylating tissue, damaging mucus, and generating free radicals [8]. Dolara et al. [9] outlined that oral antioxidants can diminish the effects and lesions induced by the DMH.

Many factors are applied to detect the severity and prognosis of colorectal cancer. CEA, as one of the markers of CRC, is a glycoprotein produced during embryonic stage until birth [10]. Despite the low serum CEA concentration, its content elevates in adults when cancer is developing [11]. PCNA, an antigen for proliferating cell nucleus, operates like a DNA clamp for the DNA polymerase in eukaryotic cells and is essential for proliferation. In the proliferating cells, more PCNA are often synthesized and expressed [12] than normal cells, which indicates an upregulated cell proliferation and is a reliable index of tumor cell proliferation [13]. Another protein that plays an important role of a brake for cell divisions is the APC [14]. Some studies have suggested the expression of PDGF, another cell division and growth factor, as a sign of the enhancing uncontrollable cell growth and tumorigenesis in cancers and tumors [15, 16]. Pan et al. [17] mentioned that this protein may cause cancer metastasis.

Royal jelly is secreted from the salivary glands of worker honeybees. The presence of the low values of this substance in the diet of female honeybees converts them into the workers, while the larvae fed an adequate level of royal jelly would develop into the queen [18]. Royal jelly (RJ) is rich in free amino acids, polypeptides, sugar, fatty acids, and minerals, the constituents of which can inhibit the tumor growth and prevent malignancies from invading. Kimura [19] showed the antitumor effect of oral royal jelly on Syrian mice. This compound regulates anticancer activity by influencing N-acetylation and inhibiting 2-aminofluorene [20]. Based on the results of the *in vitro* and *in vivo* studies, royal jelly as a medicinal agent can cope with the oxidative stress through affecting metabolic pathways and reducing free radicals [21]. Due to the great antioxidant properties of royal jelly, it becomes an ideal option for being used with other treatments such as chemotherapy [22]. The other advantages of consuming this substance in animal models are its effects on cancer factors and an increase in patients' lifespan. Furthermore, royal jelly positively affects drug-caused toxicity. Along with the decreasing inflammation and oxidative stress, being easy to access and produce can be addressed as the other benefits of utilizing this compound [23].

## 2. Method

### 2.1. Royal Jelly Preparation

Fresh royal jelly was purchased from Mazandaran province in the north of Iran (52.35° E and 36.47° N). Additionally, a part of the substance was analyzed through employing the gas chromatography-mass spectroscopy (GC-MS) technique.

### 2.2. Analysis of Royal Jelly Based on the GC-MS Technique

Royal jelly was injected into a Shimadzu GCMS-TQ8040 NX by using a microsyringe, followed by scanning for 45 minutes. The analysis process was repeated three times. Then, the components were specified by comparing their spectra with those in Wiley and NIST/EPA/NIH34-44 spectral mass libraries. The values lower than 1% were removed from the table [24].

### 2.3. Cell Culture

HT-29 cells, as a human intestinal cancer cell line, were placed in the RPMI 1640 medium with 10% fetal bovine serum (FBS) within a 75 ml flask. They were laid into a plate for cultivation after reaching the cell confluence of 75%, achieving appropriate cell morphology, and being in growth logarithmic phase. Further, trypsin-EDTA was applied to detach cells from the flask floor, and cells were counted by using the Neubauer slide [25].

### 2.4. MTT

The cells were cultivated in 96-well plates so that 7000 cells were cultured in each well. The exposure stages of royal jelly began after 48 hours, when cell frequency reached 70% in each well. Furthermore, 200 mg of royal jelly was dissolved into 500 *μ*l of DMSO, made to 2 ml volume with 1500 *μ*l of medium, and serially diluted up to 10 times. Regarding each dilution, three wells were considered for treating with royal jelly. The control (three wells) was prepared by dissolving 500 *μ*l of DMSO in 1500 *μ*l of medium and rediluting up to 10 times serially. Serial dilution was performed through mixing 1 ml of the previous solution with 1 ml of medium in each time. Following the exposure, the plates were incubated for 48 hours. Then, 5 mg/ml MTT powder was dissolved in PBS, 50 *μ*l of which was added into each well after suctioning its supernatant, and placed in an incubator for 3 hours. Finally, 150 *μ*l of DMSO was poured into each well to dissolve colored crystals. It was subjected to an ELIZA reader with the main and background wavelength of 570 and 360 nm, respectively. The results were calculated by using the Excel program, viability percentage was transferred by using the Prism software, and linear regression was determined [25].

### 2.5. Animals under Study

In the present study, 60 male Wistar rats weighing 200-210 g with the age of 8 weeks were held in the animal house of the Pasture Institute of Iran under a 12hours light/dark cycle at 20-23°C and 60-70% relative humidity. During the study, they had access to a standard level of food and water, and the intended ethical protocols were observed (IR.IAU.BABOL.REC.1399.100), alongside with the ARRIVE guidelines 2.0 [26].

### 2.6. Study Design

Vitamin E and DMH were purchased from Merck (Germany). The Wistar rats were randomly categorized into six 10-member groups. The rats in the first group exposed to no treatment and received normal saline gavage as the control group. The second and third groups received royal jelly with 300 mg/kg concentration [27] and vitamin E with 180 mg/kg dose [28] by gavage once per week, respectively. Those in the fourth group were subcutaneously injected with 30 mg/kg of DMH once a week [29]. In addition, 300 mg/kg of royal jelly and 180 mg/kg of vitamin E were, respectively, administered to the DMH-treated animals in the fifth and sixth groups.

All animals survived after the 13th week, which then were completely anesthetized intraperitoneally by using 10 mg/kg ketamine (10%, Bremer Pharma GmbH) and 80 mg/kg Xylazine (2%, AlfasanDiergeneesmiddelen BV) cocktail [30]. Following the blood sampling from the rats, colon tissue samples were collected, two parts of which were kept separately in formalin and a freezer at -80°C for histological assessment and tissue homogenate preparation, respectively.

### 2.7. Blood and Serum Tests

The blood samples were taken in two separate tubes, one of which included EDTA for CBC (Celltac Es MEK-7300 K, Nihon Kohden). Another tube without the anticoagulant were centrifuged (EBA 20, Hettich®) and utilized to measure all serum markers by using a BIOLIS24i autoanalyzer (Tokyo BoekiMedisys Inc.).

### 2.8. Colon Tissue Homogenization

To prepare tissue homogenate, 250 mg of colon tissue was homogenized in 1 ml of 50 mM phosphate buffer solution and 0.1 M EDTA with pH 7.4 in each group. Then, the mixture was centrifuged at 4°C and 12000 rpm for 20 min, the supernatant of which was isolated and held at -80°C until oxidative stress marker determination. The protein content of the homogenates was obtained by using bovine serum albumin (standard) based on the Bradford assay [31].

### 2.9. Malondialdehyde (MDA) Concentration Measurement

MDA was assessed on a TebPazhouhan Razi Kit. The tissue supernatant and reagents were allowed to reach room temperature half an hour prior to the start of the experiment. The reagents were heated up to 50°C on a bain-marie and were vortexed when any crystal was detected. The deionized water was applied to double the volume of thiobarbituric acid, which was mixed with the reagents of HOAC (×5), alkali (×10), in a 1 : 1 : 2 ratio (HOAC : alkali : thiobarbituric acid). Further, 200 *μ*l of sample (or standard) was poured into 800 *μ*l of working solution, followed by closing its lid, placing in a bain-marie at 95°C for 45 min, and cooling it in ice water containers quickly. After a 15-minute centrifugation at 3000 rpm, the samples were transferred into plate wells and their absorbance was examined at 550 nm.

### 2.10. Superoxide Dismutase (SOD) Level Determination

This stage was performed by using a SOD Activity Assay Kit (Nasdox). To evaluate SOD concentration, 50 *μ*l of the homogenate supernatant was added into sample wells, and the control received an equal volume of deionized water, to which both R1 and R2 were poured, respectively. Finally, the absorbance of the mixtures was read at 405 nm after room temperature incubation for 5 min in the absence of light.

### 2.11. Total Antioxidant Capacity (TAC)Assay

A TAC Assay Kit (Naxifer) was used for assaying a TAC level in colon tissue. Following a half-hour placement of reagents at room temperature, 2.2 ml of R2b was poured into each R2a bottle and was completely vortexed until dissolution to produce R2 solution. The R2 was mixed with an equal volume of R3 reagent and was vortexed, to which a fivefold volume of R1 was added. Regarding each well, 5 *μ*l of sample (or standard) and 250 *μ*l of working solution were, respectively, added, the optical absorbance of which was determined at 593 nm after 5 min.

### 2.12. Protein Expression by Suing Western Blot Analysis

In each group, a part of colon tissue (1 g) was frozen and lyzed with the RIPA buffer, followed by achieving tissue homogenate by using an electric homogenizer. The tissue homogenates were centrifuged at 12000 rpm for 10 min and western-blotted to obtain protein. The blots were incubated with APC, PCNA, CEA, and PDGF (primary antibodies) at 4°C for 12 hours, as well as the proper secondary antibodies related to peroxidase conjugate, respectively. This study used *β*-actin antibody as an internal control protein, relative to which the percentage of other antibodies were reported. The resultant membranes were read after washing with TBS after 10 minutes. Regarding each protein, the expression level were measured in all samples, the area under its diagram was computed by using the ImageJ software, and the ratio of this area to the *β*-actin protein was determined [25, 32].

### 2.13. Histology

In the pathological assessment, paraffin blocks (TE100, PouyaAbzarAzma) were prepared by washing colon tissues with sterile normal saline, lying in 10% formalin buffer, fixating (DS2080/H, Did Sabz Co.), dehydrating, and passaging. After cooling the blocks (TE100, PouyaAbzarAzma), the five-microns sections (DS4055, Did Sabz Co.) were H&E stained and examined by using an Olympus CX23 optical microscope. In the histological analysis, Kruskal-Wallis and Mann-Whitney *U* assays were employed for the histopathological scoring between the groups, as well as determining mitotic index to compare the significance of their differences [33, 34].

### 2.14. Data Analysis

The statistical analysis was carried out by analyzing the data of stress and inflammatory markers, CBC, serum tests, and the ratios of western blot proteins by using the SPSS 26 software based on the one-way ANOVA and Duncan's post hoc tests. The *P* value less than 0.05 was considered significant.

## 3. Results

### 3.1. Analysis of Royal Jelly through Using the GC-MS Technique

The result of GC-MS analysis indicated the existence of natural antioxidants, as well as a high value of 10-hydroxy-8-decenoic acid (10H8DA), 5-hydroxymaltol (HMT), and 5-hydroxymethylfurfural (5HM) (Table 1).

### 3.2. MTT

**Figure 1:**
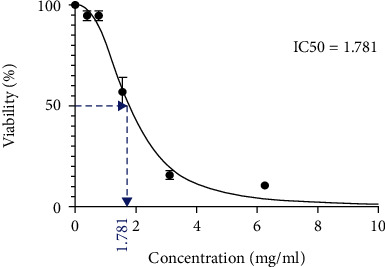
Viability level of HT-29 cells, as well as LC50. The results were read after 48 hours.

**Figure 2:**
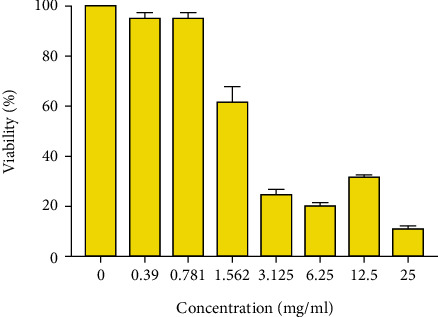
Comparison between the effects of royal jelly with various concentrations on cancer cells viability. Results are expressed as the mean ± standard error in mg/ml.

The first figure displays the cytotoxicity of royal jelly with different concentrations on HT-29 cell lines. As depicted, the cytotoxicity is maximized at 25 mg/ml, and LC50 is equal to 1.781 mg/ml after 48 hours (Figures 1 and 2).

### 3.3. Hematological Parameters

The blood profile assessment reflected less RBC, hemoglobin, RDW, MCHC, MCV, MCH, and hematocrit (HCT) level in the DMH group compared to the control group. In addition, both treatment groups experienced better status in comparison with the DMH group. No significant difference was observed among other groups (Table 2).

### 3.4. WBC

As shown in Table 3, the WBC count significantly increased in the DMH group compared to the control group (*P* < 0.05), followed by a substantial decrease among the rats in the DMH + royal jelly group. Further, the DMH group exhibits a greater neutrophil percentage and a significantly lower lymphocyte percentage (*P* < 0.05), the status of which becomes better in the DMH + royal jelly-treated group. However, the platelet level was not significantly different between the groups (Table 3).

### 3.5. Biochemical and General Serum Inflammatory Markers

The results related to the serum total protein (TP) concentration revealed that the TP content was higher in the DMH group than that of the control group, which significantly diminished after receiving the royal jelly (*P* < 0.05).

**Figure 3:**
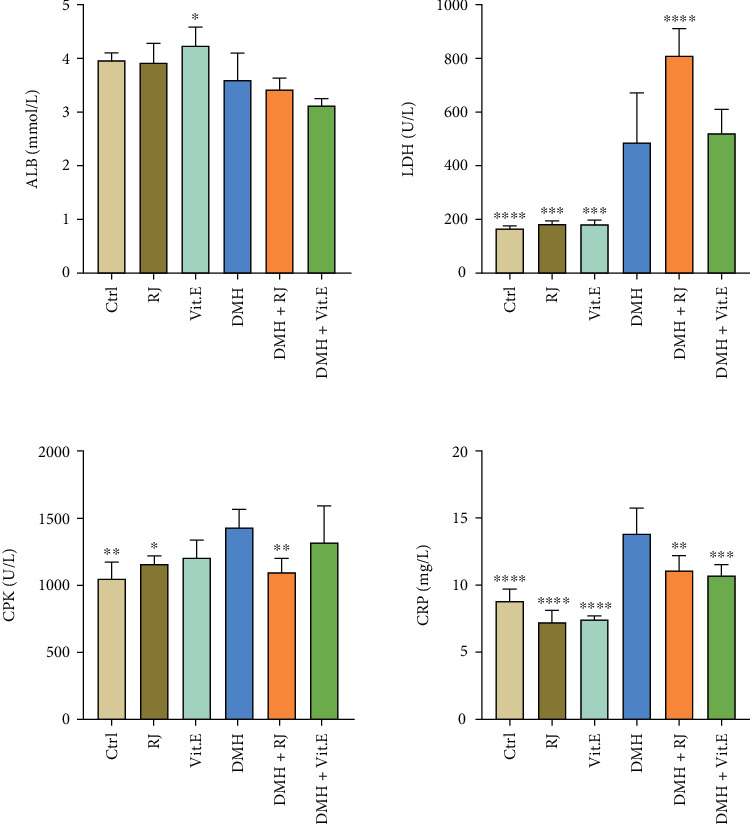
Comparison between general serum biochemical indices. ^∗^*P* < 0.05, ^∗∗^*P* < 0.01, ^∗∗∗^*P* < 0.001, and ^∗∗∗∗^*P* < 0.0001: significant compared to the DMH group. All results are expressed as the mean ± standard error. *n* = 5.

Blood albumin value was reduced by administering the DMH, but no significant difference was found between the groups (Figure 3). In addition, the results demonstrated a rise in the LDH, CRP, and CPK levels in the DMH group, in which the rise of the CRP and LDH was significant. Following the treatment with royal jelly, a decrease was seen in the CPK and CRP levels in the DMH + RJ group compared to the DMH group (Figure 3).

### 3.6. MDA Concentration in Colon Tissues

**Figure 4:**
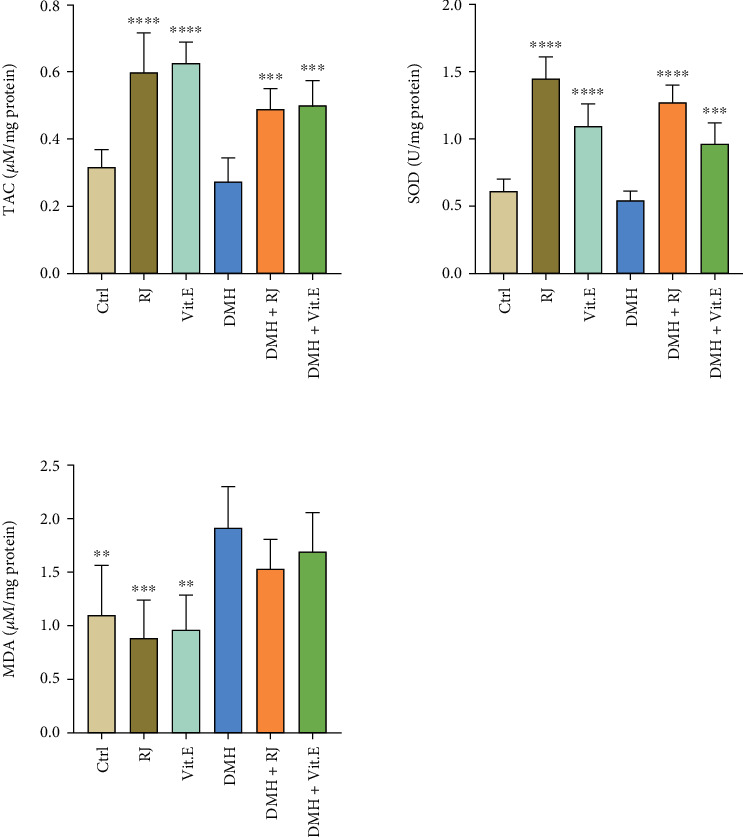
MDA, SOD, and TAC levels in the different groups. ^∗∗^*P* < 0.01, ^∗∗∗^*P* < 0.001, and ^∗∗∗∗^*P* < 0.0001: significant compared to the DMH group. All results are expressed as the mean ± standard error. *n* = 5.

The DMH group had a significantly higher MDA level than the control group (*P* < 0.01), while this value was lower in the DMH + royal jelly-exposed group (Figure 4).

### 3.7. SOD Level in Colon Tissues

The use of DMH resulted in the diminishing SOD concentration in the colon tissue compared to the control group. In this regard, a significant enhancement was obtained among the rats treated with royal jelly in comparison with the DMH group, which was a higher number than the vitamin E-receiving group (Figure 4).

### 3.8. TAC in Colon Tissues

In terms of TAC, a significant increase was found in the both treatment groups compared to the DMH group (*P* < 0.001) (Figure 4).

### 3.9. Protein Expression Level (Western Blot)

As demonstrated in Table 4, the ratio of protein expression in the groups reflects a significantly more expression of the CEA in the DMH group than that of the control group (*P* < 0.05), which is also significantly lower than that of the rats receiving DMH + royal jelly (*P* < 0.05). In the DMH group, a significantly higher level of the PCNA and PDGF was detected compared to the control group (*P* < 0.05), while they both significantly declined after treating with the royal jelly (*P* < 0.05). This improvement in status was greater in the animals treated with DMH + royal jelly than those of receiving DMH + vitamin E. Finally, the APC was significantly less expressed in the DMH group in comparison with the control group, followed by an increase in the both treatment groups with insignificant (*P* > 0.05) difference from the DMH group (Table 4).

### 3.10. Histological Observations

**Figure 5:**
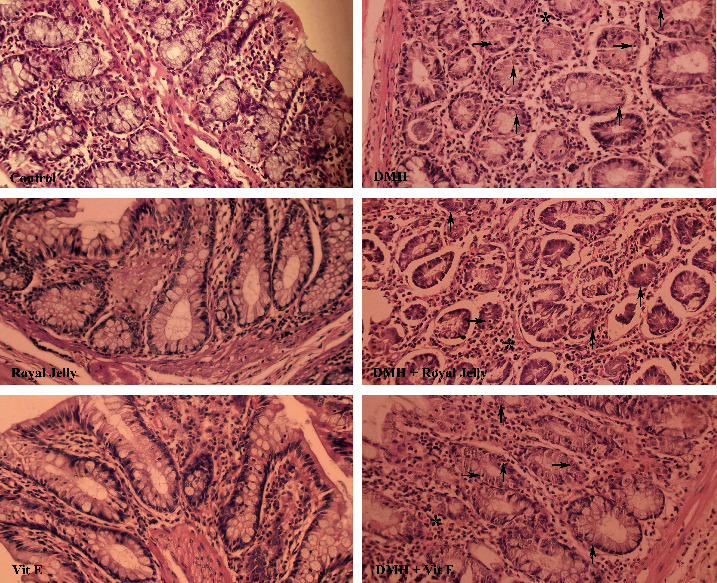
Comparison of colon tissues indices of the different groups. Normal tissue conditions in the control, royal jelly, and vitamin E groups. Mitosis, necrosis, and inflammatory cells which are illustrated with upward arrows, right arrows, and ^∗^, respectively, which can be seen in the DMH and treatment groups. ×40 magnification, H&E staining.

This figure includes scoring and comparing pathological lesions such as the mitotic index, necrosis, and inflammatory cell infiltration level between all groups (with the average number of mitoses in 10 HPF at the tumor area, [33]) (Figure 5).

The observations revealed no significant difference between the control, royal jelly, and vitamin E groups with respect to necrosis, mitosis, and inflammatory cells, although the indices of the DMH group significantly differed from those of the control (*P* < 0.01). Necrosis, mitosis, and inflammatory cells significantly were reduced among the rats exposed to DMH + royal jelly in comparison with the DMH group (*P* < 0.01). RJ consuming led to a significant diminution in the mitotic index compared to the vitamin E-receiving group (Table 5).

## 4. Discussion

The oral consumption of royal jelly exhibited anticancer activity during the pretumorigenesis phases of colorectal cancer [23]. This substance also resulted in reducing tissue stress and diminishing inflammation in precarcinogenesis stage. Considering the antitumor and anticancer properties of royal jelly, it significantly modulated the expression level of CEA, APC, PCNA, and PDGF proteins and regulated blood and serum factors positively. Besides, the administration of the RJ among the Wistar rats led to no adverse or toxic effect.

Some researchers have proposed the cytotoxicity of royal jelly on MRC-5 [35] and PC3 cell lines [36]. In the present study, this compound represented appropriate cytotoxicity in the HT-29 cell culture medium, as one of the most resistant and invasive cancer cell lines. In the MTT assay, RJ showed a proper anticancer ability with a low LC50.

The results suggested the ability of royal jelly to kill human intestinal precancerous and cancer cells, which is consistent with those of some earlier studies [20, 23, 37]. Based on the results of the *in vitro* study using MTT assay, royal jelly at 25 mg/kg concentration exhibited the highest cytotoxicity against cancer cells. The presence of the antioxidants such as 10H8DA, HMT, and 5HM in royal jelly is likely responsible for the main part of its anticancer properties [38]. HMT is a strong antioxidant and a free-radical scavenger [39], which specifically protects erythrocytes against the ROS [40] and decreases the MDA level [41]. Further, 5HM is a phenolic compound with antioxidant and anti-inflammatory effects, which reduces inflammatory cells in the sites and improves tissue stress conditions [42].

In this study, colorectal cancer was induced in rats following the use of DMH for 13 consecutive weeks. Exposing to the DMH results in the higher expression of oncogenes, disrupting the apoptosis process through the two methylation and mutation mechanisms and simultaneously enhancing the ROS value in some tissues [8]. After administering the DMH, the RBC was the first body cell which experienced a reduced stability (Table 2), since the oxidative stress is among the causes of disturbing RBC's normal level in blood [43], leading to other abnormalities and diseases such as cancer [44, 45]. Maintaining the oxidant balance in the body is considered very important, and therapeutic agents should have a high antioxidant ability to compensate for this elevation in the blood free radicals [46–49]. The promoted stress in erythrocytes disrupts the immune system [50]. Thus, the WBC count raises by revealing neoplasm in which the percentage of phagocyte increases [51, 52]. Furthermore, the lipid peroxidation causes interference in the erythrocyte function and worsens the condition through creating an oxidative stress in the cell membranes, destructing them and releasing their substances in blood [53].

Among the biomarkers that reflect the acute body status [54–56], the CPK level was enhanced in the DMH group, which may be attributed to the heart damage, myocardial microinfarctions, skeletal muscle, and/or liver problems. The significant elevation in the CRP value (*P* < 0.05) was another biomarker in this regard, which was significantly diminished by treating with DMH + royal jelly. In the DMH group, a significant improvement was found in the regard of the LDH level (*P* < 0.05), which demonstrates vast destructions at the cellular level. However, this value was reduced after consumption of the royal jelly. Similarly, the results of the previous studies suggested that the royal jelly could inhibit inflammation and upregulates cell healing [57, 58].

In the present study, the DMH led to a significant elevation in the tissue stress in colon, which is the same with that of Wei et al. [59]. In addition, the SOD, an antioxidant pathway enzyme, was declined in the DMH group compared to the control group. Also, the DMH group had a significantly higher MDA concentration than the control group (*P* < 0.05), which indicated an increase in the tissue stress, as well as the existence of the ROS metabolites [60]. So, the promoted lipid peroxidation in the colon tissue following the exposure to the DMH was one of the reasons for enhancing stress [61]. Further, an increase in the lipid peroxidation products exhibited an inflammatory and carcinogenesis effect and accelerated the secondary neoplasms, along with leading to the more oxidative stress [62]. A reduction in the TAC level in the DMH group decreased the body's ability to fight against the oxidative stress and intensified the peroxidation-caused lesions, which has the same consequences as the previous studies [63, 64]. After administering the royal jelly, a significant enhancement was observed in the TAC level compared to the DMH group (*P* < 0.05) due to its antioxidant properties. Therefore, an increase was occurred in the power of the antioxidant system in the body against the free radicals and metabolites that were generated in the lipid peroxidation cycle. This issue caused a better antioxidant balance in the body, which is consistent with the results that were obtained by Milani et al. [65] and Wielsøe et al. [66].

Furthermore, the DMH led to a various tissue lesions in the colon, which was in line with the results of Hamiza et al. [67]. Scoring and staging the lesions, based on the protocols provided in the previous studies [34, 68, 69], demonstrated that the DMH-treated rats exhibited the signs of necrosis, inflammatory cells, and a higher mitotic index, which are attributed to the methylation and increased free radicals in the tissue [70]. Compared to the DMH group, the consumption of royal jelly, similar to previous study by Nagai et al. [71], significantly diminished the mitotic index, necrosis, and inflammatory cells because of the reducing of the free radicals, which were caused by the declining lipid peroxidation in the colon tissue (*P* < 0.01), whereas they were increased by the DMH at the first place [72].

Finally, the western blot revealed that the CEA level was significantly more expressed in the DMH group compared to the control group (*P* < 0.05). This issue is related to the fact that this protein is often promoted by the cancerous cell secretions [73]. The DMH caused the occurred changes, leading to a disturbance in the tissue oxidant balance, methylation, mutation, and carcinogenesis, respectively. Accordingly, a significant rise was found in the PCNA protein expression in the groups receiving DMH in comparison to the control group (*P* < 0.05). However, royal jelly significantly decreased the PCNA level (*P* < 0.05) and prevented the cancer by inhibiting the proliferation. In fact, royal jelly represents its antitumor and anticancer activity by stopping the cancer cells from reproducing. Additionally, the declined APC protein expression caused chromosomal instability, mutation, and cancer. In the DMH group, the expression of this protein was lower than the control group (*P* < 0.05), which reflects the loss of chromosomal stability, an increase in unbridled cell divisions, and cell carcinogenesis. The rats exposed to DMH + royal jelly experienced an enhancement in the reexpression of the APC gene, leading to a greater concentration of the APC protein in the cells and more chromosomal stability, leading to a controlled cell reproduction cycle and decelerated tumorigenesis. The results revealed a significant improvement in the PDGF content of the DMH group in comparison with the control group (*P* < 0.05), followed by a significant reduction in the DMH + royal jelly-treated group because of the inhibition of this factor from being produced by consuming the royal jelly (*P* < 0.05). Similarly, Okda et al. [74] outlined that administering indomethacin + vitamin D combination significantly diminishes the elevation which was occurred in the tissue stress, PDGF, and CEA marker levels due to the exposure to DMH based on a similar technique.

## 5. Conclusion

Royal jelly can effectively reduce the oxidative stress and free radicals through controlling the pathway of lipid peroxidation and improves the power of body's antioxidant defense system significantly. This substance controls the cell division markers, which results in a decreased uncontrollable cell division, a declined rate of carcinogenesis, and a lower extent of tumorigenesis. Overall, RJ can be used as an agent for fighting the colorectal cancer due to its cytotoxicity level on cancerous cells and has a potential for raising the tissue's TAC.

## Figures and Tables

**Table 1:** Results of analyzing royal jelly by using the GC-MS method. Retention time (RT): the duration between injection (initial time) and detection.

Chemical constituents	Retention time	Peak area(%)	Molecular weight(mg/mol)	Molecular formula
5-Hydroxymaltol	8.674	5.98	142.12	C_6_H_6_O_4_
(+)-Verbenone	9.505	4.96	150.21	C_10_H_14_O
5-Hydroxymethylfurfural	10.158	18.97	126.11	C_6_H_6_O_3_
10-Hydroxy-8-decenoic acid	15.829	13.81	186.25	C_10_H_18_O_3_
(Z)-8-Tetradecenal	15.927	1.67	210.36	C_14_H_26_O
Alpha-linolenic acid	20.074	14.65	278.42	C_18_H_30_O_2_
Ostreasterol	29.967	9.04	398.7	C_28_H_46_O

**Table 2:** Comparison between RBC parameters.

Groups	RBC (×10^6^/*μ*l)	HGB (g/dl)	HCT (%)	MCV (fl)	MCH (pg)	MCHC (g/dl)	RDW (%)
Control	8.12 ± 0.22	13.64 ± 0.45	40.7 ± 1.46	51.06 ± 0.78	17.16 ± 0.34	33.56 ± 0.24	14.35 ± 0.35
Royal Jelly	8.89 ± 0.19^∗^	14.59 ± 0.33^∗^	43.59 ± 0.92	49.76 ± 0.52	16.61 ± 0.29	33.38 ± 0.21	14.08 ± 0.5
Vitamin E	8.32 ± 0.19	14.15 ± 0.27	41.2 ± 2.04	50.24 ± 0.5	17.08 ± 0.35	33.6 ± 0.48	13.76 ± 0.32
DMH	7.54 ± 0.37	12.94 ± 0.47	39.19 ± 1.78	49.43 ± 1.68	16.99 ± 0.29	32.44 ± 0.33	13.51 ± 0.31
DMH + royal jelly	8.59 ± 0.26^∗^	14.1 ± 0.45	42.28 ± 1.49	50.08 ± 0.53	16.86 ± 0.18	33.69 ± 0.21^∗^	14.03 ± 0.37
DMH + vitamin E	8.09 ± 0.24	13.24 ± 0.4	39.88 ± 1.55	49.51 ± 0.87	17.11 ± 0.24	33.59 ± 0.64	13.61 ± 0.39

^∗^ *P* < 0.05: significant compared to the DMH group. All results are expressed as the mean ± standard error. *n* = 10.

**Table 3:** Comparison between platelet and WBC parameters.

Groups	WBC (×10^3^/*μ*l)	Neutrophil (%)	Lymphocyte (%)	PLT (×10^3^/*μ*l)
Control	7.93 ± 0.44^∗^	59.08 ± 3^∗^	45 ± 3.6^∗^	734 ± 23.84
Royal Jelly	7.43 ± 0.65^∗^	59.75 ± 3.42^∗^	42.25 ± 2.35^∗^	716.75 ± 29.32
Vitamin E	7.6 ± 0.48^∗^	62.16 ± 3.64	42.21 ± 2.71^∗^	791.13 ± 23.13
DMH	9.58 ± 0.75	72.4 ± 5.05	26.93 ± 2.96	786.63 ± 51.79
DMH + royal jelly	8.34 ± 0.33	64.15 ± 4.06	34.16 ± 4.34	739.25 ± 28.55
DMH + vitamin E	8.2 ± 0.48	70.5 ± 3.18	29.71 ± 2.76	803.63 ± 32.88

^∗^ *P* < 0.05: significant compared to the DMH group. All results are expressed as the mean ± standard error. *n* = 10.

**Table 4:** Comparison between the expression levels of proteins relative to that of *β*-actin in colon tissue.

Groups	CEA	PCNA	PDGF	APC
Control	0.39 ± 0.01^∗^	0.72 ± 0.03^∗^	0.76 ± 0.02^∗^	0.99 ± 0.02^∗^
DMH	0.82 ± 0.09	1.49 ± 0.07	1.38 ± 0.05	0.65 ± 0.06
DMH + RJ	0.48 ± 0.04^∗^	1.01 ± 0.1^∗^	0.94 ± 0.04^∗^	0.80 ± 0.05
DMH + Vit E	0.71 ± 0.04	1.02 ± 0.05^∗^	0.98 ± 0.11^∗^	0.79 ± 0.06

^∗^ *P* < 0.05: significant compared to the DMH group. All results are expressed as the mean ± standard error. *n* = 5.

**Table 5:** Comparison between the types of colon tissue injuries in different inflammatory groups.

Groups	Necrosis	Inflammatory cells in filtration	Mitosis
Control	0.20 ± 0.13	0.10 ± 0.10	0.30 ± 0.15
Royal Jelly	0.10 ± 0.10	0.10 ± 0.10	0.10 ± 0.10
Vitamin E	0.10 ± 0.10	0.10 ± 0.10	0.20 ± 0.13
DMH	2.10 ± 0.23*a*, *e*, *f*	1.90 ± 0.28*a*, *e*	20.70 ± 1.08*a*, *e*, *f*
DMH + royal jelly	1.00 ± 0.26*b*, *e*	0.80 ± 0.20*b*, *e*	8.80 ± 0.89*b*, *e*, *g*
DMH + vitamin E	1.20 ± 0.25*c*, *f*	1.30 ± 0.30*c*	16.70 ± 1.21*c*, *f*, *g*

*P* values less than 0.0001 were considered statistically significant. All results are expressed as the mean ± standard error. ^a^Statistically significant differences were observed between the control and DMH groups. ^b^Statistically significant differences were observed between the control and DMH + royal jelly-exposed groups. ^c^Statistically significant differences were observed between the control and DMH + vitamin E-exposed groups. ^e^Statistically significant differences were observed between the DMH and DMH + royal jelly-exposed groups. *n* = 10.

## Data Availability

Upon request, data supporting the conclusion of our study are accessible by corresponding author.
